# Urolithin A Protects Chondrocytes From Mechanical Overloading-Induced Injuries

**DOI:** 10.3389/fphar.2021.703847

**Published:** 2021-06-17

**Authors:** Yuchen He, Lauren Yocum, Peter G Alexander, Michael J Jurczak, Hang Lin

**Affiliations:** ^1^ Department of Orthopaedic Surgery, University of Pittsburgh School of Medicine, Pittsburgh, PA, United States; ^2^ Department of Orthopaedics, Xiangya Hospital, Central South University, Changsha, China; ^3^ Division of Endocrinology and Metabolism, Department of Medicine, University of Pittsburgh School of Medicine, Pittsburgh, PA, United States; ^4^ Department of Bioengineering, University of Pittsburgh Swanson School of Engineering, Pittsburgh, PA, United States; ^5^ McGowan Institute for Regenerative Medicine, University of Pittsburgh School of Medicine, Pittsburgh, PA, United States

**Keywords:** urolithin A, cartilage, mitophagy, senescence, chondrocyte, mechanical loading

## Abstract

Physiological mechanical stimulation has been shown to promote chondrogenesis, but excessive mechanical loading results in cartilage degradation. Currently, the underlying mechanotransduction pathways in the context of physiological and injurious loading are not fully understood. In this study, we aim to identify the critical factors that dictate chondrocyte response to mechanical overloading, as well as to develop therapeutics that protect chondrocytes from mechanical injuries. Specifically, human chondrocytes were loaded in hyaluronic hydrogel and then subjected to dynamic compressive loading under 5% (DL-5% group) or 25% strain (DL-25% group). Compared to static culture and DL-5%, DL-25% reduced cartilage matrix formation from chondrocytes, which was accompanied by the increased senescence level, as revealed by higher expression of p21, p53, and senescence-associated beta-galactosidase (SA-β-Gal). Interestingly, mitophagy was suppressed by DL-25%, suggesting a possible role for the restoration mitophagy in reducing cartilage degeneration with mechanical overloading. Next, we treated the mechanically overloaded samples (DL-25%) with Urolithin A (UA), a natural metabolite previously shown to enhance mitophagy in other cell types. qRT-PCR, histology, and immunostaining results confirmed that UA treatment significantly increased the quantity and quality of cartilage matrix deposition. Interestingly, UA also suppressed the senescence level induced by mechanical overloading, demonstrating its senomorphic potential. Mechanistic analysis confirmed that UA functioned partially by enhancing mitophagy. In summary, our results show that mechanical overloading results in cartilage degradation partially through the impairment of mitophagy. This study also identifies UA’s novel use as a compound that can protect chondrocytes from mechanical injuries, supporting high-quality cartilage formation/maintenance.

## Introduction

Osteoarthritis (OA) is the most common degenerative joint disease affecting up to 240 million people around the world (Vos et al., 2016). While OA now is accepted as a disease affecting all joint elements, cartilage quality and quantity still represent a central criterion when assessing OA severity and treatment efficacy (Deveza and Loeser 2018). The exact mechanisms of OA pathogenesis remain largely unclear, but known risk factors include aging, gender, obesity, genetic predisposition, acute trauma, chronic overload, hormone profile, and metabolic syndrome (He, Li, et al., 2020; Sun et al., 2021). As a result of the limited knowledge of OA pathogenic mechanisms, there are no disease-modifying osteoarthritis drugs (DMOADs) that can halt or reverse OA progression.

Given that the main function of the knee joint is to bear weight, mechanical overloading and overuse may result in damage to cartilage, which initiates cartilage degradation and increases the likelihood of OA (Merkely et al., 2018). How these injurious mechanical cues are converted into biochemical signals and subsequently cause detrimental activities in cartilage and chondrocytes has been limited, but insightful. For example, gremlin-1 was identified as a mechanical loading-inducible factor in chondrocytes. In response to cyclic strain or hydrostatic pressure loading, elevated gremlin-1 activates the nuclear factor kappa-light-chain-enhancer of activated B cells(NF-**K**B), leading to catabolic enzyme induction and subsequent cartilage degradation (Chang et al. 2019, Tong et al. 2015). Other mechanisms involved in overloading-induced OA progression include primary cilia destruction, mitochondrial dysfunction, and impaired autophagy (He, Makarczyk, et al. 2020, He et al. 2016, Tong et al. 2019, Zheng et al. 2019).

Mitochondrion is a semi-autonomous organelle surrounded by a double membrane. In addition to energy generation, mitochondria has emerged as key participants in sensing and integrating cues from the environment to trigger adaptive and compensatory responses in cells (Galvan et al., 2017). Dysfunctional mitochondria are identified by decreased mitochondrial membrane potential, increased proton leak and increased generation of ROS (Chapman et al., 2019). Increased level of PTEN induced kinase 1 (PINK1), parkin, and lysosome related proteins are also indicators of mitochondrial malfunction (López de Figueroa et al., 2015). Since the accumulation of damaged mitochondria is often observed in OA chondrocytes, removal of damaged mitochondria to restore overall mitochondrial quality and function has been suggested as a new target for DMOAD development (Mao et al., 2020). Mitophagy is a type of autophagy that selectively degrades damaged mitochondria, preventing dysfunctional mitochondria accumulation and thus protecting cells from cellular degeneration (Palikaras et al., 2018). Defective mitophagy is thought to be associated with apoptosis, aging, and a range of pathological processes, including arthritis and disc degeneration (Hu et al., 2020). However, exactly how mitochondrial function and mitophagy are modulated by mechanical loading is unclear and the interaction between mitochondrial function and mechanotransduction-relevant molecules in the context of mechanical damaging-induced cartilage degradation remains unknown.

Urolithin A (UA), a natural dietary, microflora-derived metabolite resulting from ellagitannin transformation by gut bacteria (Muku et al., 2018), has been shown to induce mitophagy and thus prevent dysfunctional mitochondria accumulation (Ryu et al., 2016). A recent clinical study indicated that UA was biosafe and improved mitochondrial and cellular health (Andreux et al., 2019). Moreover, intra-articular UA injection was shown to reduce OA severity in animal models (Ding S-l et al., 2020), in which UA primarily functioned by suppressing inflammation. Whether UA is able to target other OA-relevant pathways, such as restoration of mitophagy in OA chondrocytes, is unreported.

In this study, it was hypothesized that 1) excessive mechanical loading causes dysfunctional mitochondria accumulation, increases cellular senescence and inflammation level, and impairs chondrogenic potential of chondrocytes; 2) UA treatment can restore mitophagy in mechanically injured chondrocytes, thus preserving their capacity in generating high-quality cartilage. To test the hypotheses, we first established an *in vitro* mechanical injury-induced cartilage degradation model. Specifically, human chondrocytes were encapsulated in hyaluronic acid (HA) scaffold and subjected to mechanical loading with 5% or 25% strains for 14 days. As the first step towards understanding chondrocyte responses to mechanical signals, we used this model to analyze level changes in molecules associated with chondrogenesis, cartilage degradation, mitochondrial function, cellular senescence, and inflammation. Next, UA was supplemented into culture medium and its ability to protect mechanically injured chondrocytes was examined. Lastly, we conducted a mechanistic study to understand the molecular pathways influenced by UA treatment.

## Materials and Methods

### Cell Isolation and Expansion

With the approval from the University of Pittsburgh Committee for Oversight of Research and Clinical Training Involving Decedents (CORID), cartilage tissues were harvested from the knee joints of deidentified donors without joint disease. To isolate chondrocytes, fresh articular cartilage tissues were rinsed with dissection medium containing high glucose Dulbecco's Modified Eagle Medium (DMEM, Gibco/Thermo Fisher Scientific, Waltham, MA, United States) containing 2 × Antibiotics-Antimycotics (Life Technologies, Carlsbad, CA, United States) and cut into ∼1-mm^3^ pieces and digested in 10 ml/g wet weight cartilage dissection medium with collagenase type II (Worthington Biochemical Corporation, Lakewood, NJ, United States) at 1 mg/ml (w/v) for 16 h in a shaker at 37°C. The mixture was then passed through a 70 μm cell strainer to collect single chondrocytes. The isolated chondrocytes were seeded in tissue-culture flasks at a density of 1 × 10^4^ cells/cm^2^ and maintained in growth medium (GM, DMEM containing 10% fetal bovine serum (FBS, Life Technologies) and 1% Antibiotics-Antimycotics). After cells were fully attached to the culture substrate (usually after 7 days), the medium was changed every three days until cells reached 70–80% confluency. The cells were then detached with Trypsin/EDTA (Gibco/Thermo Fisher Scientific) and passaged. Passage 1 (P1) chondrocytes from six donors were pooled (Supplementary Table S1). P2 chondrocytes were used in all experiments.

### Hydrogel Synthesis and Cell Encapsulation

**FIGURE 1:**
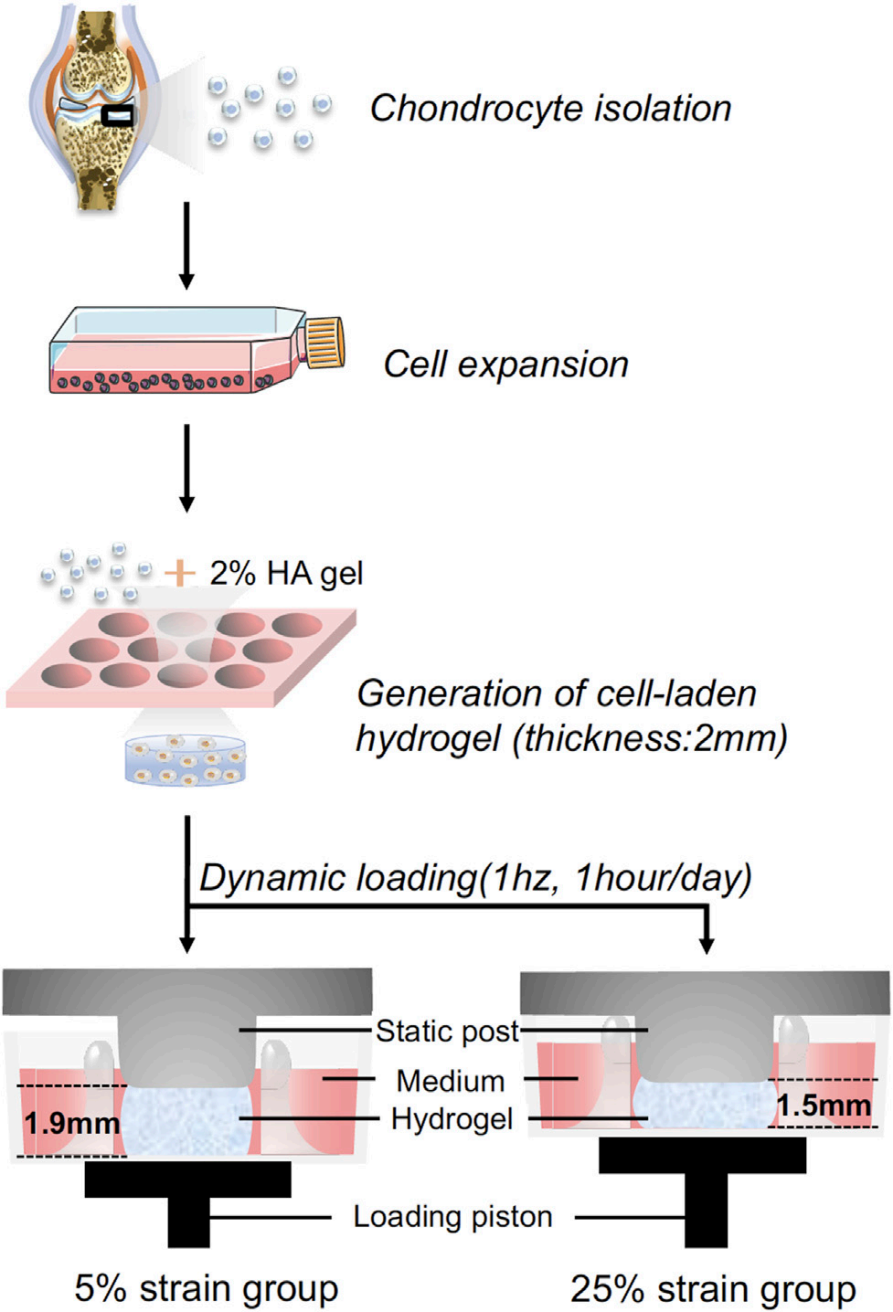
Schematic illustration of the experimental process. Human chondrocytes were isolated from healthy cartilage, expanded to passage 2, and then loaded in hyaluronic acid (HA) hydrogel. The cell-laded constructs were placed between the static post and loading piston, and then subjected to dynamic loading. Through controlling the travel distance of the piston, cyclic loading at 5% or 25% strain was achieved.

The photoinitiator, lithium phenyl-2,4,6-tri-methylbenzoylphosphinate (LAP), was synthesized as previously reported (Lin et al., 2014). The methacrylated hyaluronic acid (HA, Advanced BioMatrix, CA, United States) was dissolved in phosphate-buffered saline (PBS) at a concentration of 2% (w/v) with 0.15% LAP added as the photoinitiator. As shown in Figure 1, pooled P2 human chondrocytes were suspended in the 2% HA solution at 20 × 10^6^ cells/ml. The suspension was pipetted into a silicone mold with 6 mm (D) × 2 mm (H) cylindrical void space and photocrosslinked for 2 min using a flashlight (395 nm wavelength, 7w, LED Wholesaler, Hayward, CA, United States). Constructs were cultured in chondrogenic medium overnight in NUNC 6-well plates that were not treated for cell culture (Thermo Fisher Scientific, Waltham, MA, United States). These cell-loaded hydrogels were subjected to the dynamic loading over the next day for 14 days.

### Mechanical Loading

Dynamic compressive loading was performed in a Mechano-Active Transduction and Evaluation (MATE) bioreactor (Apex Biomedical LLC, Wilsonville, OR, United States) (Figure 1). Dynamic cyclic compression was applied to cell-loaded gels with controlled strain and displacement within a tissue culture incubator (37°C, 5% CO_2_) (Iseki et al., 2019). To mimic a cadence of daily walking, the MATE system was operated at 1 Hz with different strains (5 and 25%) for 1 h per day over 14 consecutive days. All samples were maintained in 3 ml chondrogenic medium [CM: high-glucose DMEM supplemented with 1% Antibiotics-Antimycotics, 40 μg/mL L-proline (Sigma, St. Louis, MO, United States), 10 μg/ml ITS+ (Thermo Fisher Scientific), 50 μg/ml ascorbate 2-phosphate, 10 ng/ml transforming growth factor (TGF)-β3 (Peprotech, Rocky Hill, NJ, United States)]. Medium for all groups was changed every 3 days. The non-loaded samples in static culture were used as the control. Chondrogenesis was analyzed after 14 days.

### RNA Isolation and Quantitative Real-Time Polymerase Chain Reaction (qRT-PCR)

Samples were washed with PBS twice. An electric pestle was applied to crash the gels. Cells were released from the gels and homogenized in Qiazol (Qiagen, Germantown, MD, United States). Total RNA was isolated and purified using an RNeasy Plus Universal Mini Kit (Qiagen, Cat. No. 74104) according to the manufacturer’s protocol. The reverse transcription to the complementary DNA (cDNA) was completed using SuperScript IV VILO Master Mix (Invitrogen, Waltham, MA, United States). qRT-PCR was performed on a real-time PCR instrument (QuantStudio 3, Applied Biosystems, Foster City, CA, United States) using the SYBR Green Reaction Mix (Applied Biosystems) with custom primers ordered from Integrated DNA Technologies (IDT, Newark, NJ, United States). Relative gene expression levels were calculated through the 2^−ΔΔCt^ method. Ribosomal protein L13A (*RPL13A*) was used as the housekeeping gene. Full names and abbreviations of genes and their corresponding proteins are listed in Supplementary Table S2. Primer sequences are listed in Supplementary Table S3.

### Safranin O/Fast Green Staining

Safranin O/Fast green staining was used to assess glycosaminoglycans (GAGs) content. Samples were fixed in 10% buffered formalin phosphate solution (Fisher Chemical, Fair Lawn, NJ, United States) overnight and dehydrated with an increasing ethanol gradient. After being soaked in xylene for 2 h and then in liquid paraffin overnight, samples were embedded in paraffin. The blocks were sectioned at 6 μm thickness using the RM 2255 Fully Automated Rotary Microtome (Leica Biosystems, Buffalo Grove, IL, United States). Slides were dried and subjected to Safranin O/Fast green counterstaining (Sigma-Aldrich, St. Louis, MO, United States). Cell nuclei were stained with Hematoxylin solution (Sigma-Aldrich).

### Immunohistochemistry Staining (IHC)

Samples were prepared and sectioned as described in *Safranin O/Fast Green Staining* section. After rehydration in a decreasing alcohol gradient and distilled water, slides were blocked with 10% horse serum (Vector Labs, Burlingame, CA, United States) in PBS for 1 h and then incubated overnight at 4°C with a primary antibody. All antibodies used in this study were listed in Supplementary Table S4. Afterward, the sections were sequentially incubated with biotinylated secondary antibodies for 30 min, and then horseradish peroxidase (HRP)-conjugated streptavidin for another 30 min using the Vectastain Elite ABC-HRP Kit (Vector Labs). Lastly, peroxidase substrate (NovaRed substrate kit) was added for various time periods appropriate for different antigenic targets. After counterstaining with hematoxylin, slides were dehydrated, mounted, and cover-slipped.

### Senescent Associated β-Galactosidase Staining (SA-β-Gal Staining)

After being fixed with 4% Paraformaldehyde aqueous solution (Gibco/Thermo Fisher Scientific), samples were dehydrated with increasing gradient sucrose solutions and then frozen in Cryo-Gel (LeicaBiosystems, Richmond, IL, United States). Cryosectioning was done using the Leica CM1850 Cryostat (Mercedes Scientific, Lakewood Ranch, FL, United States) at 12 μm thickness. Senescence β-Galactosidase Staining Kit (BioVision, Milpitas, CA, United States) was used to assess β-galactosidase activity according to the manufacturer’s instructions.

### Western Blot

Samples were washed with pre-cold PBS for three times. A pestle was used to homogenize the gels. Total proteins were extracted by suspending the homogenate in RIPA buffer (Sigma-Aldrich) supplemented with protease and phosphatase Inhibitor Single-Use Cocktail (Gibco/Thermo Fisher Scientific) and then centrifuging at 10,000 g at 4°C for 10 min. Protein concentration of the supernatant was determined by the Pierce™ BCA Protein Assay Kit BCA kit (Thermo Scientific). The samples were diluted in RIPA as required for equal loading, mixed with Laemmli buffer (Bio-Rad, Hercules, CA, United States), and then denatured at 95°C for 5 min. Proteins were fractionated electrophoretically on the NuPAGE 4–12%, Bis-Tris Mini Protein Gel (Gibco/Thermo Fisher Scientific) and then transferred to a polyvinylidene fluoride (PVDF) membrane using the iBlot Dry Blotting System (Invitrogen). The membrane was blocked with 3% non-fat milk (BioRad Hercules, CA, United States), diluted with TBST (0.1% Tween 20 (Sigma-Aldrich) in 1 × Tris buffered saline (TBS, Gibco/Thermo Fisher Scientific) at room temperature for 1 h, washed, and incubated with a primary antibody at 4 °C overnight on a rotating shaker. Next, the membrane was washed 5 times with TBST buffer and incubated with horseradish peroxidase (HRP)-linked secondary antibodies (GE Healthcare Life Sciences, Malborough, MA, United States) for 1.5 h at room temperature. After being washed 5 times with TBST, the membrane was incubated with the chemiluminescence substrate SuperSignal West Dura Extended Duration Substrate (Thermo Fisher Scientific). Images were acquired using the ChemiDocTM Touch Imaging System (Bio-Rad, Hercules, CA, United States).

### Drug Testing

Urolithin A, 3,8-Dihydroxy-6H-benzo [c]chromen-6-one (UA, Sigma-Aldrich), was dissolved in Dimethyl Sulfoxide (DMSO, Sigma-Aldrich) at 20 mg/ml to make a stock. To optimize dosage, chondrocyte-laded scaffolds were cultured in chondrogenic medium containing 1 ng/ml interleukin-1β (IL-1 β, PeproTec, company information) and assigned to four groups. UA was added into the medium of different groups with the final concentration of 0, 1, 10, and 100 μM, respectively. After 7 days, samples were collected for PCR and western blot analysis.

We used ImageJ Analysis Toolbox to conduct deconvolution and downstream analysis of semi-quantitative histological staining and western blotting results. After the background color pixels were eliminated, we selected and calculated the integrated density (ImtDen) of the remaining color pixels of histological images using the statistical color model. Automatic Nuclei Segmentation was implemented for the detection of positively stained nuclei in DAB stained images. The pre-defined parameters included a window size of 25 × 25 pixels (half size of nuclei), a minimum size constraint of 100 pixels, and a final size constraint of 150 pixels. For each type of protein and staining, we analyzed three WB bands or three slides of each experimental group.

### UA Treatment

Medium for the UA treatment group was CM with 10 µM UA. The medium for the control group was CM only. Medium for the experimental groups was changed every 3 days. Using the method described in *Mechanical Loading* section, dynamic cyclic compression was applied to all groups at 1 Hz with 25% strains for 1 h per day over 14 consecutive days.

### Statistical Analysis

We applied GraphPad Prism eight for comparation. Quantitative data were expressed as mean ± standard deviations (S.D.). Statistics were analyzed by *t*-test for two-group comparison, and One-way or two-way analysis of variance (ANOVA) for multi-comparison between groups. We used Tukey's post hoc multiple comparisons test as the posttest method for ANOVA. *p* values < 0.05 were considered statistically significant.

## Results

### Dynamic Loading Under 5 and 25% Strain Displays Beneficial and Detrimental Influences on Chondrogenesis, Respectively

**FIGURE 2:**
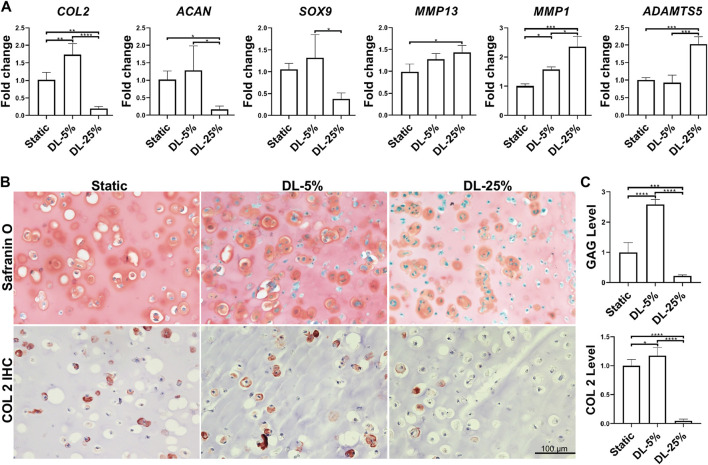
Influence of dynamic loading on chondrogenesis. **(A)** Relative gene expression levels of representative anabolic and catabolic genes. Dynamic compressive loadings with 5% (DL-5%) and 25% (DL-25%) strains were used. Data were normalized to that from the non-loaded static culture group (Static) (set as 1); *N* = 4. **(B)** Safranin O/Fast green staining and COL2 immunohistochemistry (IHC) to assess the deposition of cartilage matrix. Scale bar: 100 µm. **(C)** Based on the staining density, the levels of GAGs and COL2 were semi-quantitated using ImageJ software. *N* = 3. *p < 0.05; **p < 0.01; ***p < 0.001; ****p < 0.0001.

The experimental process is shown in Figure 1. Human chondrocytes were encapsulated within 2% HA hydrogel and then subjected to dynamic compressive loading at a strain of 5% or 25%. After 14 days of chondrogenic culture, samples were collected and analyzed. As shown in Figure 2A, dynamic loading at 5% strain (DL-5%) resulted in significantly higher expression of *COL2* when compared to static culture without compression (Static group)*.* A similar trend was observed in *ACAN* and *SOX9* expression, although no significant difference was observed. Safranin O/Fast green staining and COL2 IHC staining further indicated that DL-5% resulted in more cartilage matrix production from human chondrocytes than static culture (Figures 2B,C).

When the magnitude of dynamic compressive loading strain increased to supraphysiological 25% (DL-25% group), anabolic gene expression levels were reduced, which concomitantly accoupled increased catabolic gene expression, such as *MMP1, MMP13, and ADAMTS5* (Figure 2A)*.* Compared to the other two groups, samples from the DL-25% group displayed a significantly less histologically evident sGAG protein and COL2 protein deposition, suggesting impaired chondrogenesis (Figures 2B,C).

### Dynamic Loading Under 25% Strain Results in Significantly Increased Senescence Level

**FIGURE 3:**
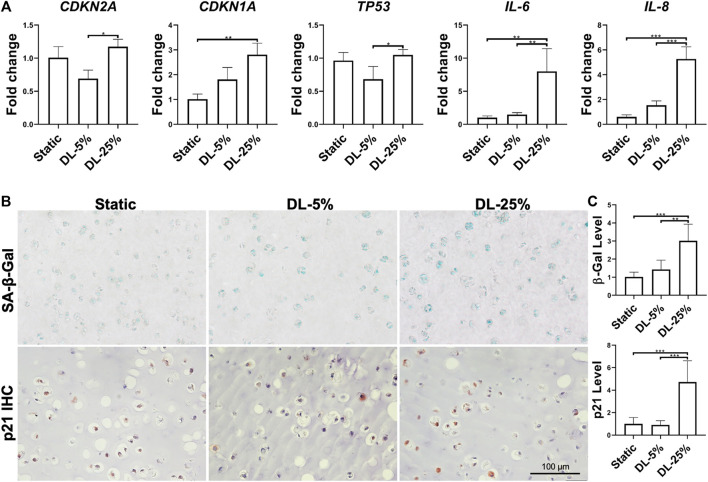
Assessment of dynamic loading on the levels of cellular senescence and inflammation. **(A)** Relative gene expression levels of representative senescence and inflammation-associated markers. Data were normalized to that from the Static culture group (set as 1). *N* = 4. **(B)** SA-β-Gal staining, p21 and NF-кB p65 IHC for samples from different groups. Scale bar: 100 µm. **(C)** Based on staining density, the levels of SA-β-Gal and p21 were semi-quantitated using ImageJ software. *N* = 3. *p < 0.05; **p < 0.01; ***p < 0.001; ****p < 0.0001.

The extent of cellular senescence and inflammation after dynamic loading was assayed by examining gene expression and protein levels. Compared to the other two groups, cells in cartilage created under DL-25% expressed more *CDKN1A*, *TP53*, *IL-6*, and *IL-8* as well generated higher levels of SA-β-Gal, p21 and NF-κB p65 (Figure 3 and Supplementary Figure S1)*.* We did not observe noticeable p16 level changes in response to dynamic loading.

Taken together, DL-25% significantly impaired cartilage formation from human chondrocytes, which also reduced neo-cartilage quality by upregulating the degradation, inflammation, and senescence-relevant molecules.

### Dynamic Loading Influences the Expression Levels of Factors That Are Associated With Chondrogenesis, Senescence and Mitochondrial Function

**FIGURE 4:**
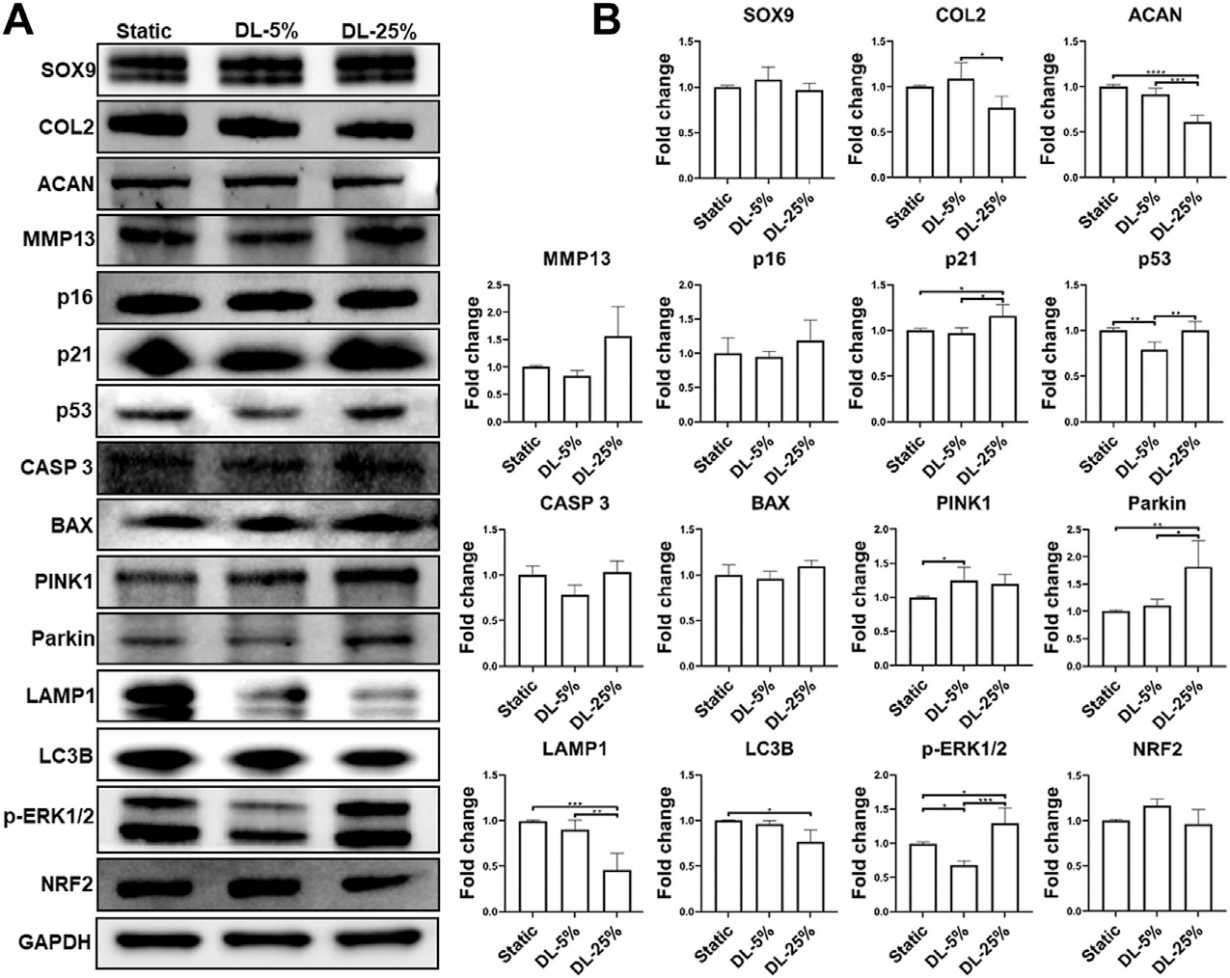
Examination of molecules that were regulated by mechanical loading. Chondrocyte-laden scaffolds were maintained in Static culture or dynamic compressive loadings with 5% (DL-5%) or 25% (DL-25%) strains for 14 days. **(A)** Western blot with GAPDH as the loading control (B) Relative protein levels normalized to GAPDH were semi-quantitated using ImageJ software. *N* = 3. *p < 0.05; **p < 0.01; ***p < 0.001; ****p < 0.0001.

To understand the mechanism underlying chondrocyte response to mechanical loading, samples cultured under three conditions were assessed by western blot analysis of molecules known to be relevant with chondrogenesis, degradation, apoptosis, and senescence (Figure 4). We also examined the levels of factors associated with mitochondrial function. Consistent with the observation above, the DL-25% group had reduced COL2 & ACAN levels and increased MMP13 level, confirming its detrimental effect on chondrogenesis. p21 and p53 were also upregulated upon DL-25% treatment. We did not observe alternation of p16 protein level. Bcl-2-associated X protein (BAX) and caspase 3 (CASP 3) protein levels were noticeably increased in the DL-25% groups, but the difference was not statistically significant in the semi-quantitative analysis.

Regarding the association between mechanical loading and mitochondrial function, levels of microtubule-associated proteins 1A/1B light chain 3B (LC3B) and Lysosomal-associated membrane protein 1 (LAMP1), markers of autophagy, were found to be significantly reduced upon DL-25% treatment, which indicates the impaired capacity of cells in removing damaged mitochondria. In contrast, Parkinson’s disease-associated protein (Parkin) and PTEN-induced kinase 1 (PINK1) were upregulated when DL-25% was applied, suggesting a protective action in response to mechanical stress-induced mitochondrial dysfunction. Interestingly, phosphorylated ERK1/2 (*p*-ERK1/2) level was downregulated under DL-5%, but upregulated under DN-25%. The level of nuclear factor erythroid 2-related factor 2 (NRF2), a protein that regulates the expression of antioxidant proteins, decreased in the DL-25% group. Lastly, DL-25% results in a significant increase of NF-κB p65 level (Supplementary Figure S1). The findings collectively indicated that DL-25% impaired chondrogenesis and mitophagy, which also caused cellular stress and increased the tread toward apoptosis. In fact, an increased level of damaged mitochondria was observed in the DL-25% group (Supplementary Figure S2).

### Urolithin A at 10 µM Suppresses IL-1β Induced Injuries to Chondrocytes in 3D Culture

**FIGURE 5:**
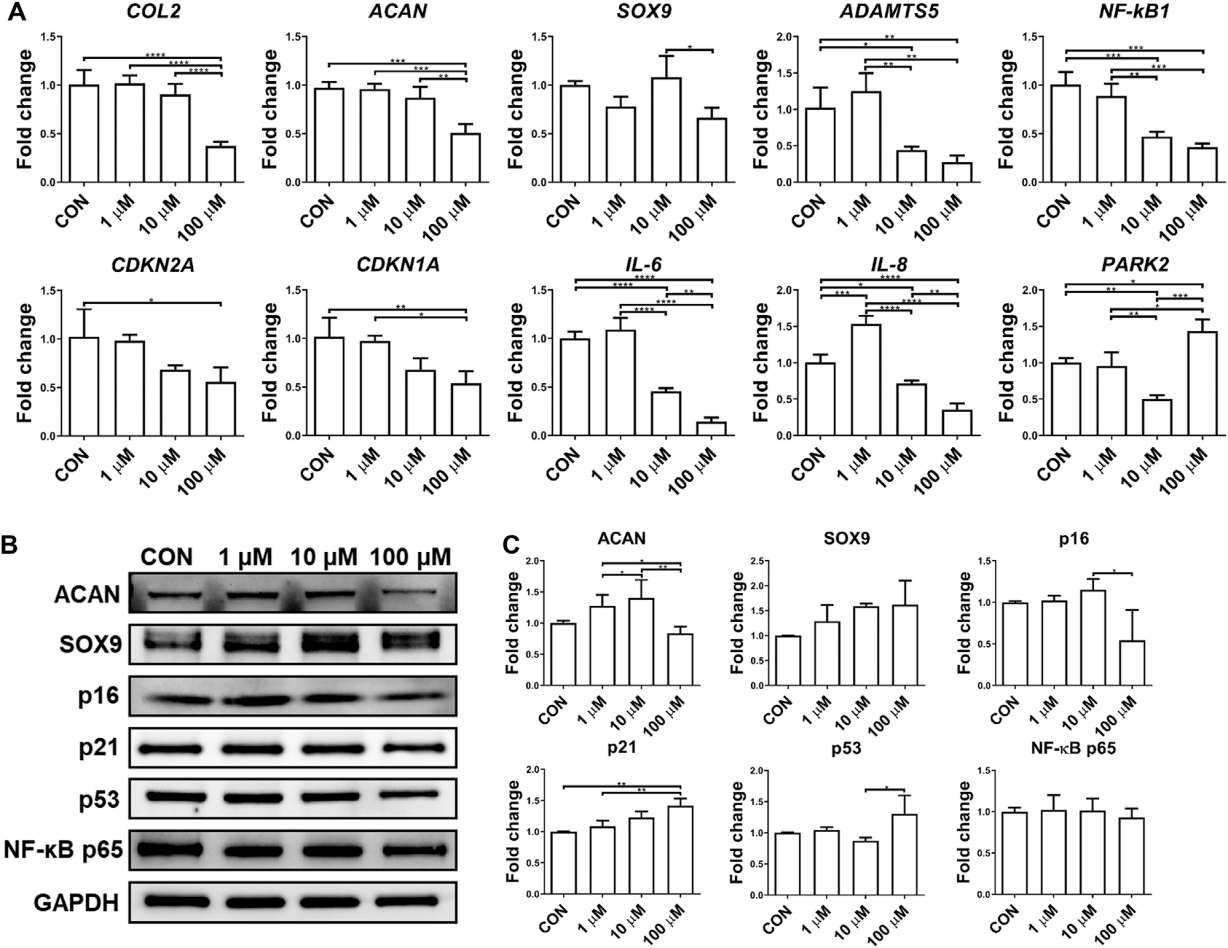
Effect of UA treatment on chondrocytes-laden scaffold challenged by IL-1β. Constructs were treated with IL-1β only (CON), or co-treated with 1, 10, or 100 μM UA. **(A)** qRT-PCR analysis to examine the expression levels of representative chondrogenesis, cellular senescence, and inflammation-relevant genes. Data were normalized to that from the untreated control group (CON) (set as 1). **(B)** Western blot and **(C)** semi-quantitative analysis for assessing protein levels using ImageJ software. *N* = 3; *p < 0.05; **p < 0.01; ***p < 0.001; ****p < 0.0001.

Although the anti-inflammatory potential of UA has been reported on chondrocyte monolayer culture, it was not validated in 3D culture. In this study, we thus examined the influence of UA treatment on chondrocytes cultured in scaffolds. As shown in Figure 5, UA did not promote the expression of chondrogenic genes in chondrocytes. At 100 μM, UA was even detrimental to chondrogenesis. As expected, UA reduced the expression of pro-inflammatory cytokines and cartilage-degrading enzymes, which may function through suppressing NF-κB (Figures 5A,B). Interestingly, we did not observe changes in p16, p21, and p53 levels after UA treatment. Since UA at 10 µM displayed anti-inflammatory capacity without affecting chondrogenesis, this dose was selected for the following studies.

### Urolithin A Treatment Protects Chondrocytes From the Damage Caused by DL-25%

**FIGURE 6:**
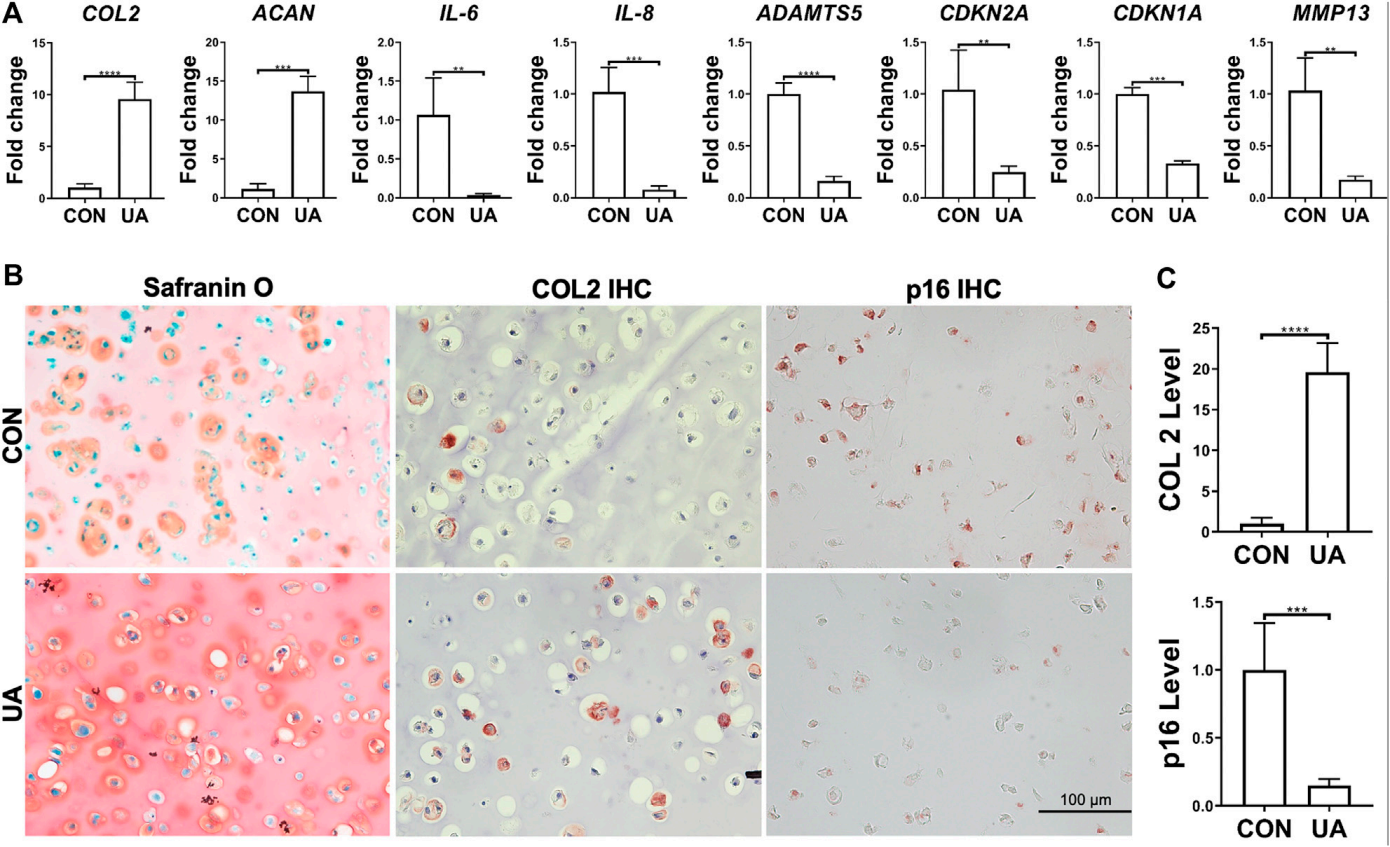
UA treatment on the chondrocytes-laden scaffold that was subjected to mechanical overloading (DL-25%). **(A)** Relative expression levels of representative anabolic and catabolic genes after UA treatment (UA). Data were normalized to that from the untreated control group (CON) (set as 1), in which samples were subjected to DL-25% without UA treatment. *N* = 4. **(B)** Safranin O/Fast green staining, COL2 and p16 IHC. Scale bar: 100 µm. **(C)** Based on staining density, the levels of COL2 and p16 were semi-quantitated using ImageJ. *N* = 3. *p < 0.05; **p < 0.01; ***p < 0.001; ****p < 0.0001.

UA’s effect on chondrocytes, under injurious conditions induced by DL-25%, was evaluated. Compared to the control without being treated with UA (CON group), the experimental group with UA treatment significantly inhibited DL-25%-induced injures to chondrocytes, revealed by higher *COL2* and *ACAN* expression levels and lower *ADAMTS5*, *MMP-13, IL-6, IL-8, CDKN1A*, *and CDKN2A* expression levels (Figure 6A). Results from COL2 & p16 IHC and Safranin O/Fast green staining indicated that UA not only promoted cartilage matrix deposition, but also increased cartilage quality by suppressing senescence (Figures 6B,C).

### Urolithin A Displays Chondro-Supportive, Anti-senescent, Anti-inflammatory, and Mitophagy-Enhancing Potentials

**FIGURE 7:**
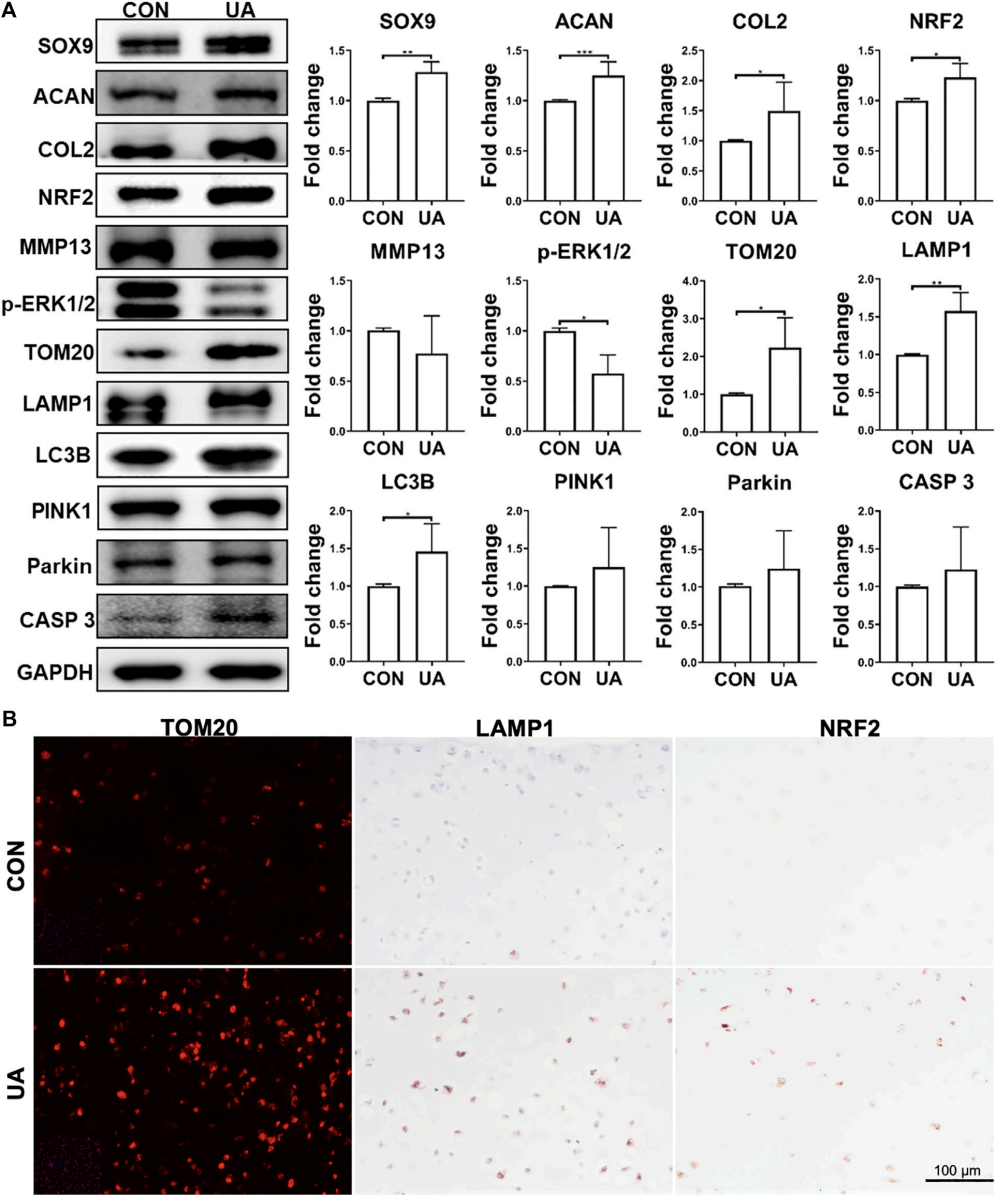
Protein levels in samples with (UA) or without (CON) UA treatment **(A)** Representative western blot and semi-quantitative analysis of protein levels. *N* = 3. **(B)** Representative images from immunofluorescence or IHC for TOM2O, LAMP1 and NRF2. Scale bar: 100 µm. *N* = 3. *p < 0.05; **p < 0.01; ***p < 0.001; ****p < 0.0001.

Western blot was performed to assess selective protein expression levels (Figure 7A). UA treatment enhanced SOX9, ACAN, and COL2 levels, which was consistent with its chondro-promotive function observation above. TOM20, LAMP1, LC3B, and CASP 3 levels increased after UA treatment, implying the restoration of previously suppressed mitophagy and functional mitochondrial by DL-25%. UA also significantly downregulated *p*-ERK1/2. Moreover, the antioxidative protein, NRF2, was upregulated after UA treatment. Interestingly, no statistically significant difference was observed regarding PINK1 and Parkin expression levels. But, the increasing trend indicated that UA treatment was related to the up-regulated mitophagy level. We also used IF and IHC staining to examine TOM20, LAMP1 and NRF2 levels (Figure 7B), and results implied that UA elevated mitochondrial function as well as increased capacity against oxidative damage triggered by mechanical injury. Lastly, we did not observe an influence of UA treatment on p65 level (Supplementary Figure S3).

**FIGURE 8:**
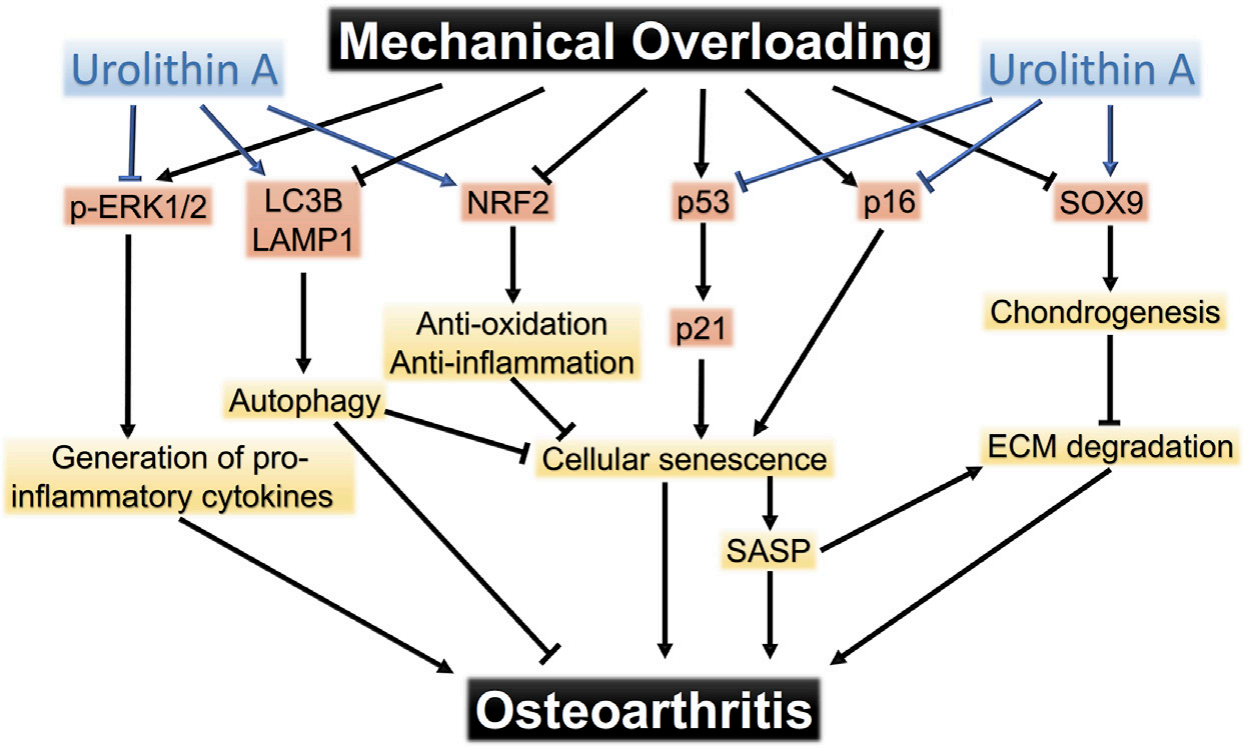
The pathways that mediate mechanical overloading-induced damage to chondrocytes. Urolithin A protects chondrocytes from mechanical injuries through targeting multiple pathways.

On the basis of the results above, we proposed the pathways that mediated chondrocyte response to mechanical overloading (Figure 8). The potential mechanism of UA treatment on these signaling pathways was also shown.

## Discussion

Physiological mechanical stimulation is critical to chondrocytes in maintaining extracellular matrix homeostasis (Chen et al., 2016). However, excessive mechanical loading induces apoptosis or stimulates inflammatory cascades within the tissue (Jørgensen et al., 2017), which has been recognized as an OA risk factor. Understanding how injurious loading contributes to cartilage degradation and OA development has thus been attracting research interests for years. In this study, we found damaging dynamic loading results in increased cellular senescence, elevated inflammation, reduced mitochondrial quality, and impaired mitophagy in chondrocytes, which together leads to a poor cartilage formation. We also demonstrated that UA, a mitophagy activator, is able to partially reverse the inferior phenotype in chondrocytes resulting from supraphysiological loading through targeting multiple signaling pathways.

To model the *in vivo* biomechanical environment, cyclic tensile system, osmotic loading system, hydrostatic pressure loading system, and load/strain-controlled cyclic loading system have been previously used (Samvelyan et al., 2020; Fu et al., 2021). In this study, we aimed to develop a 3D hydrogel scaffold to perform dynamic loading that mimics cartilage compression during daily activity. Data from healthy human knee joints shows that the overall compressive strain of tibial cartilage increases in a nonlinear fashion with walk duration. Starting from 10 min of walking to 40 min, the overall compressive strain increases from 2 ± 1% to 6 ± 8% (Paranjape et al., 2019). Though chondrocytes laying in different layers of cartilage respond differently to the compressive loading, it is believed that loading strain below 5% represents the physiological situation as walking while strain above 20% exceeds the normal range (Madden et al., 2013). As for the frequency choice, data from the Multicenter Osteoarthritis Study (MOST) showed that the range of moderate daily walking steps is 6,000 to 7,900 (Voinier et al., 2020), indicating that the cartilage in each knee joint bears 3,000 to 4,000 compressions per day. Thus, we selected a condition that resulted in 3,600 times of loading per day in this study. Regarding the loading intensity, parameters vary in different studies. In general, cyclic compression at frequencies of 0.01–1 Hz, with strain amplitudes of 1–5%, is beneficial to *in vitro* chondrogenesis (Griffin et al., 2005), which was further confirmed in our study.

To create an injurious mechanical loading, there is a discrepancy between studies. Quiroga *et al.* observed that local cartilage degradation started when it was exposed to compression with 10% strain, and 40% collagen fibers were damaged when loading strain was above 15% (Párraga Quiroga et al., 2017). However, Patwari *et al.* showed that compression with 65% strain was needed to induce injuries to human osteochondral plugs (Patwari et al., 2007). In other studies, strains ranging from 10 to 35% created mechanical damage on cartilage (Henao-Murillo et al., 2019). The differences in conditions and outcomes were partially due to the difference in the models being used. In this study, mechanical loading with 25% strain was sufficient to induce a markedly reduced chondrogenesis within HA hydrogel. It should be noted that the beneficial or detrimental influence of loading on cells also highly depends on the cell environment, such as native cartilage vs. engineered cartilage, or scaffolds with different physical and biochemical properties (Raghunath et al., 2007).

With the establishment of physiological and damaging loading conditions, we next examined the underlying mechanism responsible for converting mechanical cues into biochemical signals. As the major transcription factor that dictates chondrogenesis, SOX9 level in chondrocytes is remarkably reduced in the articular cartilage of osteoarthritis patients (Ouyang et al., 2019). In this study, though mechanical loading seemed to affect the *SOX9* gene expression, the protein level remained unchanged when compared to the non-loaded control group. This may be due to TGF-β presence in chondrogenic medium during loading, which was supplemented to maintain chondrocyte phenotype and promote cartilage matrix synthesis. It is well known that TGF-β significantly promotes the *SOX9* upregulation (Coricor and Serra 2016). The active TGF-β concentration (10 ng/ml) used exceeded normal physiological levels (Albro et al., 2012). Therefore, mechanical loading’s effect on SOX9 level was most likely masked by TGF-β.

Chondrocyte senescence is now considered an important feature in OA cartilage (Coryell et al., 2021). However, whether compressive loading can directly induce senescence phenotype generation in chondrocytes is rarely reported. Hallmarks of senescent cells included increased p53, p21, and p16 expression, enhanced SA-β-Gal generation, upregulated MMP and ADAMTS expression, and increased pro-inflammatory cytokine secretion, such as IL-6, IL-17, and IL-1β (Hernandez-Segura et al., 2018). Based on our results, these senescent hallmarks were observed in the excessive loading group, thus providing, for the first time, direct evidence of compressive overloading inducing chondrocyte senescence. Recently, the mechanically sensitive ion channel, transient receptor potential vanilloid 4 (TRPV4), was shown to mediate mechanical loading-induced inflammation, which could also participate in generating senescent phenotype (Nims et al., 2020). Of note, we did not observe a p16 level change in response to the mechanical stimuli. Similar results were reported in a study that tested cyclic mechanical tension on the nucleus pulposus cells (Feng et al., 2018), in which the condition that resulted in 20% elongation of cells led to senescent phenotype, revealed by elevated SA-β-Gal staining. However, p16 expression was not affected. This inconsistency between unchanged p16 level and increased senescence may be due to p16’s multiple roles in different cellular processes. Besides, a recent study showed that the effects of chondrocyte senescence on OA are more likely driven by the senescence-associated secretory phenotype (SASP) factors than by the loss of replicative function in chondrocytes, and that p16 is not essential for SASP production (Diekman et al., 2018). Therefore, p16’s exact role in cartilage degradation and OA pathogenesis requires further investigation.

Similar to p16, p21 is another senescence marker (Xiong et al., 1993). As a major target of p53, p21 is activated in response to a variety of cellular stresses, such as DNA damage, resulting in cell-cycle arrest, senescence, and apoptosis to prevent the proliferation of damaged cells (Benson et al., 2014). Cyclic mechanical overloading is believed to cause oxidant-dependent mitochondrial dysfunction in chondrocytes (Coleman et al., 2016). This mitochondrial dysfunction leads to the accumulation and release of lethal amounts of reactive oxygen species (ROS), resulting in DNA damage and cell death (Brouillette et al., 2014). Therefore, it is not surprising that p53/p21 was activated and upregulated in the DL-25% group, which was consistent with another study that used cyclic mechanical tension to create cellular senescence (Feng, Yang, Zhang, Lan, Huang, Liu and Zhou 2018).

Preclinical studies using chondrocyte cultures and animal models have provided evidence that mitochondrial dysfunction participates in OA onset and progression by increasing ROS production (Collins et al., 2018). Increased ROS level disrupts homeostatic signaling and promotes OA via modifying proteins and lipids, damaging DNA, inducing catabolic changes, and eventually resulting in cell death (Arra et al., 2020). NRF2 is a stress response protein that protects chondrocytes from oxidative damage, hyperoxia, and endoplasmic reticulum stress by inducing cytoprotective and antioxidant gene expression and elevating intracellular glutathione levels (Trachootham et al., 2008). Decreased NRF2 levels were observed in the DL-25% group, indicating that mechanical overloading hindered NRF2’s antioxidant mechanism in chondrocytes. Thus, upregulating the NRF2 level represents a reasonable approach to OA disease modification (Xue et al., 2017). In this study, UA treatment increased NRF2 levels (Figure 7), indicating its potential application in OA treatment.

In addition to impairing cell antioxidative defense, excessive loading also increases ROS via directly causing mitochondrial dysfunction (Delco et al., 2018). PINK1 is a mitochondrial serine/threonine-protein kinase, which accumulates on the outer membrane of damaged mitochondria (Lin and Kang 2008). However, there are inconsistent conclusions regarding the effects of PINK1 in cells. Wang *et al.* reported that PINK1 played a protective role by clearing damaged mitochondrial and alleviating cell senescence under oxidative stress (Wang et al., 2018). However, Shen H.J. *et al.* found that PINK1 mediated mitophagy in chondrocytes, contributing to cartilage degeneration in OA (Shin et al., 2019). Besides, compression-induced PINK1/Parkin-mediated mitophagy might not be a protective response (Huang et al., 2020). In our study, UA, the mitophagy activator, did not affect PINK1 and Parkin levels.

In contrast, LC3B and LAMP1 levels were regulated by UA treatment. LC3B, a common autophagosome marker, is a central protein in the autophagy pathway, functioning in autophagy substrate selection and autophagosome biogenesis (Sun et al., 2020). LAMP1 is a lysosomal membrane protein, which is responsible for maintaining lysosomal integrity, pH, catabolism, and forming autophagy-lysosomal organelles (Eskelinen 2006). Compared to the static group, DL-25% dramatically suppressed LC3B and LAMP1 expression, which is believed to have inhibited autophagic vacuole formation (Cheng et al., 2018). UA treatment significantly elevated LC3B and LAMP1 expression, thus restoring autophagy and dysfunctional mitochondria clearance.

Currently, therapeutic regimens applied in today's clinical OA management are only partially effective and cannot reverse the process underlying OA. Senolytics and senomorphics are two promising therapeutic classes that alleviate aging-associated pathologies. Senolytics are a class of small molecules that can selectively induce senescent cell death and improve health in humans (Childs et al., 2015). Instead of killing cells, senomorphics are small molecules that repress SASP by inhibiting protein activity related to inflammation, or inhibiting the production of SASP factors such as IL-6 (Coryell, Diekman and Loeser 2021). Chondrocytes are the unique cellular component of adult human articular cartilage responsible for matrix component turnover, therefore maintaining healthy chondrocytes is important for cartilage health. Thus, senomorphics may be more beneficial than senolytics in OA treatment by enhancing cell quality rather than removing cells entirely.

UA is an active metabolite of polyphenol ellagic acid, resulting from ellagitannin transformation by specific intestinal flora. In addition to its antisenescence, antioxidant, anticancer, anti-inflammatory, and antimicrobial properties (Espín et al., 2013; Liu et al., 2019), UA is well-tolerated, non-toxic, and has no adverse effects following dietary intake (Heilman et al., 2017; Andreux, Blanco-Bose, Ryu, Burdet, Ibberson, Aebischer, Auwerx, Singh and Rinsch 2019), making it an attractive DMOAD candidate. Currently, two studies have reported UA use for OA treatment in animal models, and both studies only focused on its anti-inflammatory potential (Fu et al., 2019; Ding SL. et al., 2020). In this study, it was reported for the first time that, UA’s protective effect on overloading-induced cartilage damage. Moreover, our results revealed UA's new function on anti-senescence and mitochondrial homeostasis maintenance. Considering its demonstrated capacity in reducing SASP, UA may represent a novel senomorphic for OA treatment.

Our *in vitro* mechanical loading model is advantageous for controlling the cartilage loading history and examining mechanisms and biochemical factors that affect cartilage metabolism. Still, there are some disadvantages. First, although cartilage degradation is the major feature (Peters et al., 2018), OA is now widely accepted as a whole-joint disease. In this study, we only investigated chondrocyte responses to mechanical loading. The crosstalk between cartilage and other joint components such as subchondral bone, synovium, and other joint elements needs to be further addressed. Particularly, the recently emerging microphysiological system can be adopted to study mechanical loading and UA treatment in the context of the "whole joint" (Makarczyk et al., 2021). Second, 14-days loading is still a relatively short investigation time compared to a mammal’s life span. Particularly, OA is a chronic disease, and long-term assessment will be more informative for future clinical trials. Lastly, though we identified several molecules participating in the regulation of chondrocyte response to mechanical loading, whether a direct causal relationship exists is still missing. In the future, methods that can manipulate protein levels, such as gene silencing, are needed to further confirm their roles in mechanotransduction.

Our study highlights several findings that have important implications for understanding the intricate relationship between mechanical loading and OA progression. Specifically, it is concluded that 1.) moderate loading, under 5% strain, displays beneficial influences on chondrogenesis; 2.) excessive loading, under 25% strain, results in mitochondrial dysfunction, ROS accumulation, senescence, ECM degradation, and inflammation in chondrocytes; 3.) UA represents a novel senomorphic and DMOAD, that alleviates and reverses overloading-induced chondrocyte damage by targeting multiple signaling pathways.

## Data Availability

The original contributions presented in the study are included in the article/Supplementary Material, further inquiries can be directed to the corresponding author.

## References

[B1] Albro M. B. Cigan A. D. Nims R. J. Yeroushalmi K. J. Oungoulian S. R. Hung C. T. (2012). Shearing of Synovial Fluid Activates Latent TGF-β. Osteoarthritis and Cartilage 20, 1374–1382. 10.1016/j.joca.2012.07.006 22858668 PMC3448789

[B2] Andreux P. A. Blanco-Bose W. Ryu D. Burdet F. Ibberson M. Aebischer P. (2019). The Mitophagy Activator Urolithin A Is Safe and Induces a Molecular Signature of Improved Mitochondrial and Cellular Health in Humans. Nat. Metab. 1, 595–603. 10.1038/s42255-019-0073-4 32694802

[B3] Arra M. Swarnkar G. Ke K. Otero J. E. Ying J. Duan X. (2020). LDHA-mediated ROS Generation in Chondrocytes Is a Potential Therapeutic Target for Osteoarthritis. Nat. Commun. 11 (11), 3427–3442. 10.1038/s41467-020-17242-0 32647171 PMC7347613

[B4] Benson E. K. Mungamuri S. K. Attie O. Kracikova M. Sachidanandam R. Manfredi J. J. (2014). p53-dependent Gene Repression through P21 Is Mediated by Recruitment of E2F4 Repression Complexes. Oncogene (33), 3959–3969. 10.1038/onc.2013.378 24096481 PMC4067464

[B5] Brouillette M. J. Ramakrishnan P. S. Wagner V. M. Sauter E. E. Journot B. J. McKinley T. O. (2014). Strain-dependent Oxidant Release in Articular Cartilage Originates from Mitochondria. Biomech. Model. Mechanobiol 13, 565–572. 10.1007/s10237-013-0518-8 23896937 PMC3940668

[B6] Chang S. H. Mori D. Kobayashi H. Mori Y. Nakamoto H. Okada K. (2019). Excessive Mechanical Loading Promotes Osteoarthritis through the Gremlin-1–NF-Κb Pathway. Nat. Commun. 10, 1442–1455. 10.1038/s41467-019-09491-5 30926814 PMC6441020

[B7] Chapman J. Fielder E. Passos J. F. (2019). Mitochondrial Dysfunction and Cell Senescence: Deciphering a Complex Relationship. FEBS Lett. 593, 1566–1579. 10.1002/1873-3468.13498 31211858

[B8] Chen Z. Yan F. Lu Y. (2016). The Function of Mechanical Loading on Chondrogenesis. Front. Biosci. (Landmark Ed. 21 (21), 1222–1232. 10.2741/4452 27100502

[B9] Cheng X.-T. Xie Y.-X. Zhou B. Huang N. Farfel-Becker T. Sheng Z.-H. (2018). Revisiting LAMP1 as a Marker for Degradative Autophagy-Lysosomal Organelles in the Nervous System. Autophagy 14, 1472–1474. 10.1080/15548627.2018.1482147 29940787 PMC6103665

[B10] Childs B. G. Durik M. Baker D. J. van Deursen J. M. (2015). Cellular Senescence in Aging and Age-Related Disease: from Mechanisms to Therapy. Nat. Med. 21, 1424–1435. 10.1038/nm.4000 26646499 PMC4748967

[B11] Coleman M. C. Ramakrishnan P. S. Brouillette M. J. Martin J. A. (2016). Injurious Loading of Articular Cartilage Compromises Chondrocyte Respiratory Function. Arthritis Rheumatol. 68, 662–671. 10.1002/art.39460 26473613 PMC4767543

[B12] Collins J. A. Diekman B. O. Loeser R. F. (2018). Targeting Aging for Disease Modification in Osteoarthritis. Curr. Opin. Rheumatol. Jan 30, 101–107. 10.1097/bor.0000000000000456 PMC5886778 28957964

[B13] Coricor G. Serra R. (2016). TGF-β Regulates Phosphorylation and Stabilization of Sox9 Protein in Chondrocytes through P38 and Smad Dependent Mechanisms. Sci. Rep. 6 (6), 38616–38627. 10.1038/srep38616 27929080 PMC5144132

[B14] Coryell P. R. Diekman B. O. Loeser R. F. (2021). Mechanisms and Therapeutic Implications of Cellular Senescence in Osteoarthritis. Nat. Rev. Rheumatol. 17, 47–57. 10.1038/s41584-020-00533-7 33208917 PMC8035495

[B15] Delco M. L. Bonnevie E. D. Bonassar L. J. Fortier L. A. (2018). Mitochondrial Dysfunction Is an Acute Response of Articular Chondrocytes to Mechanical Injury. J. Orthop. Res. 36, 739–750. 10.1002/jor.23882 28696002 PMC5764818

[B16] Deveza L. A. Loeser R. F. (2018). Is Osteoarthritis One Disease or a Collection of many? Rheumatology (Oxford) 57, iv34–iv42. 10.1093/rheumatology/kex417 29267932 PMC6251697

[B17] Diekman B. O. Sessions G. A. Collins J. A. Knecht A. K. Strum S. L. Mitin N. K. (2018). Expression of p16^(INK) (4a)^ Is a Biomarker of Chondrocyte Aging but Does Not Cause Osteoarthritis. Aging Cell 17, e12771. 10.1111/acel.12771 29744983 PMC6052464

[B18] Ding S-l. Pang Z-y. Chen X-m. Li Z. Liu X-x. Zhai Q-l. (2020a). Urolithin a Attenuates IL-1β-induced Inflammatory Responses and Cartilage Degradation via Inhibiting the MAPK/NF-κB Signaling Pathways in Rat Articular Chondrocytes. J. Inflamm. 17, 1–13. 10.1186/s12950-020-00242-8 PMC7092521 32210738

[B19] Ding S. L. Pang Z. Y. Chen X. M. Li Z. Liu X. X. Zhai Q. L. (2020b). Urolithin a Attenuates IL-1β-induced Inflammatory Responses and Cartilage Degradation via Inhibiting the MAPK/NF-κB Signaling Pathways in Rat Articular Chondrocytes. J. Inflamm. 17, 17–30. 10.1186/s12950-020-00242-8 PMC7092521 32210738

[B20] Eskelinen E.-L. (2006). Roles of LAMP-1 and LAMP-2 in Lysosome Biogenesis and Autophagy. Mol. Aspects Med. 27, 495–502. 10.1016/j.mam.2006.08.005 16973206

[B21] Espín J. C. Larrosa M. García-Conesa M. T. Tomás-Barberán F. (2013). Biological Significance of Urolithins, the Gut Microbial Ellagic Acid-Derived Metabolites: the Evidence So Far. Evid. Based Complement. Alternat Med. 2013, 270418. 10.1155/2013/270418 23781257 PMC3679724

[B22] Feng C. Yang M. Zhang Y. Lan M. Huang B. Liu H. (2018). Cyclic Mechanical Tension Reinforces DNA Damage and Activates the P53-P21-Rb Pathway to Induce Premature Senescence of Nucleus Pulposus Cells. Int. J. Mol. Med. 41, 3316–3326. 10.3892/ijmm.2018.3522 29512682 PMC5881642

[B23] Fu S. Meng H. Inamdar S. Das B. Gupta H. Wang W. (2021). Activation of TRPV4 by Mechanical, Osmotic or Pharmaceutical Stimulation Is Anti-inflammatory Blocking IL-1β Mediated Articular Cartilage Matrix Destruction. Osteoarthritis and Cartilage 29, 89–99. 10.1016/j.joca.2020.08.002 33395574 PMC7799379

[B24] Fu X. Gong L.-F. Wu Y.-F. Lin Z. Jiang B.-J. Wu L. (2019). Urolithin A Targets the PI3K/Akt/NF-Κb Pathways and Prevents IL-1β-induced Inflammatory Response in Human Osteoarthritis: *In Vitro* and *In Vivo* Studies. Food Funct. 10, 6135–6146. 10.1039/c9fo01332f 31497826

[B25] Galvan D. L. Green N. H. Danesh F. R. (2017). The Hallmarks of Mitochondrial Dysfunction in Chronic Kidney Disease. Kidney Int. 92, 1051–1057. 10.1016/j.kint.2017.05.034 28893420 PMC5667560

[B26] Griffin T. M. Guilak F. ss reviews. (2005). The Role of Mechanical Loading in the Onset and Progression of Osteoarthritis. Exerc. Sport Sci. Rev. 33, 195–200. 10.1097/00003677-200510000-00008 16239837

[B27] He Y. Li Z. Alexander P. G. Ocasio-Nieves B. D. Yocum L. Lin H. (2020). Pathogenesis of Osteoarthritis: Risk Factors, Regulatory Pathways in Chondrocytes, and Experimental Models. Biology (9), 194–227. 10.3390/biology9080194 32751156 PMC7464998

[B28] He Y. Makarczyk M. J. Lin H. (2020). Role of Mitochondria in Mediating Chondrocyte Response to Mechanical Stimuli. Life Sci. 263, 118602–118680. 10.1016/j.lfs.2020.118602 33086121 PMC7736591

[B29] He Z. Leong D. J. Zhuo Z. Majeska R. J. Cardoso L. Spray D. C. (2016). Strain-induced Mechanotransduction through Primary Cilia, Extracellular ATP, Purinergic Calcium Signaling, and ERK1/2 Transactivates CITED2 and Downregulates MMP-1 and MMP-13 Gene Expression in Chondrocytes. Osteoarthritis and Cartilage 24, 892–901. 10.1016/j.joca.2015.11.015 26687824

[B30] Heilman J. Andreux P. Tran N. Rinsch C. Blanco-Bose W. (2017). Safety Assessment of Urolithin A, a Metabolite Produced by the Human Gut Microbiota upon Dietary Intake of Plant Derived Ellagitannins and Ellagic Acid. Food Chem. Toxicol. 108, 289–297. 10.1016/j.fct.2017.07.050 28757461

[B31] Henao-Murillo L. Pastrama M. I. Ito K. van Donkelaar C. C. (2019). The Relationship between Proteoglycan Loss, Overloading-Induced Collagen Damage, and Cyclic Loading in Articular Cartilage. Cartilage 15, 1947603519885005. 10.1177/1947603519885005 PMC8721617 31729263

[B32] Hernandez-Segura A. Nehme J. Demaria M. (2018). Hallmarks of Cellular Senescence. Trends Cel Biol. 28, 436–453. 10.1016/j.tcb.2018.02.001 29477613

[B33] Hu S. Zhang C. Ni L. Huang C. Chen D. Shi K. (2020). Stabilization of HIF-1α Alleviates Osteoarthritis via Enhancing Mitophagy. Cell Death Dis 11, 481–497. 10.1038/s41419-020-2680-0 32587244 PMC7316774

[B34] Huang D. Peng Y. Li Z. Chen S. Deng X. Shao Z. (2020). Compression‐induced Senescence of Nucleus Pulposus Cells by Promoting Mitophagy Activation via the PINK1/PARKIN Pathway. J. Cel Mol Med 24, 5850–5864. 10.1111/jcmm.15256 PMC7214186 32281308

[B35] Iseki T. Rothrauff B. B. Kihara S. Sasaki H. Yoshiya S. Fu F. H. (2019). Dynamic Compressive Loading Improves Cartilage Repair in an *In Vitro* Model of Microfracture: Comparison of 2 Mechanical Loading Regimens on Simulated Microfracture Based on Fibrin Gel Scaffolds Encapsulating Connective Tissue Progenitor Cells. Am. J. Sports Med. 47, 2188–2199. 10.1177/0363546519855645 31307219 PMC6637720

[B36] Jørgensen A. E. M. Kjær M. Heinemeier K. M. (2017). The Effect of Aging and Mechanical Loading on the Metabolism of Articular Cartilage. J. Rheumatol. Apr 44, 410–417. 10.3899/jrheum.160226 28250141

[B37] Lin H. Cheng A. W.-M. Alexander P. G. Beck A. M. Tuan R. S. (2014). Cartilage Tissue Engineering Application of Injectable Gelatin Hydrogel with *In Situ* Visible-Light-Activated Gelation Capability in Both Air and Aqueous Solution. Tissue Eng. A 20, 2402–2411. 10.1089/ten.tea.2013.0642 PMC4161187 24575844

[B38] Lin W. Kang U. J. (2008). Characterization of PINK1 Processing, Stability, and Subcellular Localization. J. Neurochem. 106, 464–474. 10.1111/j.1471-4159.2008.05398.x 18397367 PMC3638740

[B39] Liu C.-f. Li X.-l. Zhang Z.-l. Qiu L. Ding S.-x. Xue J.-x. (2019). Antiaging Effects of Urolithin A on Replicative Senescent Human Skin Fibroblasts. Rejuvenation Res. 22, 191–200. 10.1089/rej.2018.2066 30215291

[B40] López de Figueroa P. Lotz M. K. Blanco F. J. Caramés B. (2015). Autophagy Activation and protection from Mitochondrial Dysfunction in Human Chondrocytes. Arthritis Rheumatol. 67, 966–976. 10.1002/art.39025 25605458 PMC4380780

[B41] Madden R. Han S.-K. Herzog W. (2013). Chondrocyte Deformation under Extreme Tissue Strain in Two Regions of the Rabbit Knee Joint. J. Biomech. 46, 554–560. 10.1016/j.jbiomech.2012.09.021 23089458

[B42] Makarczyk M. J. Gao Q. He Y. Li Z. Gold M. S. Hochberg M. C. (2021). Current Models for Development of Disease-Modifying Osteoarthritis Drugs. Tissue Eng. Part. C Methods. 27(2), 124–138. 10.1089/ten.TEC.2020.0309 33403944 PMC8098772

[B43] Mao X. Fu P. Wang L. Xiang C. (2020). Mitochondria: Potential Targets for Osteoarthritis. Front. Med. (Lausanne) 7, 581402. 10.3389/fmed.2020.581402 33324661 PMC7726420

[B44] Merkely G. Ackermann J. Lattermann C. (2018). Articular Cartilage Defects: Incidence, Diagnosis, and Natural History. Oper. Tech. Sports Med. 26, 156–161. 10.1053/j.otsm.2018.06.008

[B45] Muku G. E. Murray I. A. Espín J. C. Perdew G. H. (2018). Urolithin A Is a Dietary Microbiota-Derived Human Aryl Hydrocarbon Receptor Antagonist. Metabolites. Nov 29 (8), 86–104. 10.3390/metabo8040086 PMC6315438 30501068

[B46] Nims R. J. Pferdehirt L. Ho N. B. Savadipour A. Lorentz J. Sohi S. (2020). A Synthetic Mechanogenetic Gene Circuit for Autonomous Drug Delivery in Engineered Tissues. 0692–0694. 10.1126/sciadv.abd9858 PMC7840132 33571125

[B47] Ouyang Y. Wang W. Tu B. Zhu Y. Fan C. Li Y. (2019). Overexpression of SOX9 Alleviates the Progression of Human Osteoarthritis *In Vitro* and *In Vivo* . Dddt Vol. 13, 2833–2842. 10.2147/dddt.s203974 PMC6698167 31496660

[B48] Palikaras K. Lionaki E. Tavernarakis N. (2018). Mechanisms of Mitophagy in Cellular Homeostasis, Physiology and Pathology. Nat. Cel Biol 20, 1013–1022. 10.1038/s41556-018-0176-2 30154567

[B49] Paranjape C. S. Cutcliffe H. C. Grambow S. C. Utturkar G. M. Collins A. T. Garrett W. E. (2019). A New Stress Test for Knee Joint Cartilage. Sci. Rep. 9, 2283. 10.1038/s41598-018-38104-2 30783146 PMC6381136

[B50] Párraga Quiroga J. M. Wilson W. Ito K. van Donkelaar C. C. (2017). The Effect of Loading Rate on the Development of Early Damage in Articular Cartilage. Biomech. Model. Mechanobiol 16, 263–273. 10.1007/s10237-016-0815-0 27514541 PMC5285418

[B51] Patwari P. Cheng D. M. Cole A. A. Kuettner K. E. Grodzinsky A. J. (2007). Analysis of the Relationship between Peak Stress and Proteoglycan Loss Following Injurious Compression of Human post-mortem Knee and Ankle Cartilage. Biomech. Model. Mechanobiol 6, 83–89. 10.1007/s10237-006-0037-y 16715319 PMC2706506

[B52] Peters A. E. Akhtar R. Comerford E. J. Bates K. T. (2018). The Effect of Ageing and Osteoarthritis on the Mechanical Properties of Cartilage and Bone in the Human Knee Joint. Sci. Rep. 8 (8), 5931. 10.1038/s41598-018-24258-6 29651151 PMC5897376

[B53] Raghunath J. Rollo J. Sales K. M. Butler P. E. Seifalian A. M. (2007). Biomaterials and Scaffold Design: Key to Tissue-Engineering Cartilage. Biotechnol. Appl. Biochem. 46, 73–84. 10.1042/BA20060134 17227284

[B54] Ryu D. Mouchiroud L. Andreux P. A. Katsyuba E. Moullan N. Nicolet-dit-Félix A. A. (2016). Urolithin a Induces Mitophagy and Prolongs Lifespan in *C. elegans* and Increases Muscle Function in Rodents. Nat. Med. 22, 879–888. 10.1038/nm.4132 27400265

[B55] Samvelyan H. J. Hughes D. Stevens C. Staines K. A. (2020). Models of Osteoarthritis: Relevance and New Insights. Calcif Tissue Int., 1. 10.1007/s00223-020-00670-x PMC8403120 32062692

[B56] Shin H. J. Park H. Shin N. Kwon H. H. Yin Y. Hwang J. A. (2019). Pink1-mediated Chondrocytic Mitophagy Contributes to Cartilage Degeneration in Osteoarthritis. J. Clin. Med. 8, 8. 10.3390/jcm8111849 PMC6912334 31684073

[B57] Sun A. R. Udduttula A. Li J. Liu Y. Ren P.-G. Zhang P. (2021). Cartilage Tissue Engineering for Obesity-Induced Osteoarthritis: Physiology, Challenges, and Future Prospects. J. orthopaedic translation 26, 3–15. 10.1016/j.jot.2020.07.004 PMC7773977 33437618

[B58] Sun K. Jing X. Guo J. Yao X. Guo F. (2020). Mitophagy in Degenerative Joint Diseases. Autophagy Sep 24, 1–11. 10.1080/15548627.2020.1822097 PMC8496714 32967533

[B59] Tong W. Geng Y. Huang Y. Shi Y. Xiang S. Zhang N. (2015). *In Vivo* Identification and Induction of Articular Cartilage Stem Cells by Inhibiting NF-Κb Signaling in Osteoarthritis. Stem Cells 33, 3125–3137. 10.1002/stem.2124 26285913

[B60] Tong W. Zeng Y. Chow D. H. K. Yeung W. Xu J. Deng Y. (2019). Wnt16 Attenuates Osteoarthritis Progression through a PCP/JNK-mTORC1-PTHrP cascade. Ann. Rheum. Dis. 78, 551–561. 10.1136/annrheumdis-2018-214200 30745310

[B61] Trachootham D. Lu W. Ogasawara M. A. Valle N. R.-D. Huang P. (2008). Redox Regulation of Cell Survival. Antioxid. Redox Signaling 10, 1343–1374. 10.1089/ars.2007.1957 PMC2932530 18522489

[B62] Voinier D. Neogi T. Stefanik J. J. Guermazi A. Roemer F. W. Thoma L. M. (2020). Using Cumulative Load to Explain How Body Mass index and Daily Walking Relate to Worsening Knee Cartilage Damage over Two Years: the MOST Study. Arthritis Rheumatol. 72, 957–965. 10.1002/art.41181 31785075 PMC8020569

[B63] Vos T. Allen C. Arora M. Barber R. M. Bhutta Z. A. Brown A. (2016). Global, Regional, and National Incidence, Prevalence, and Years Lived with Disability for 310 Diseases and Injuries, 1990-2015: a Systematic Analysis for the Global Burden of Disease Study 2015. Lancet 388, 1545–1602. 10.1016/S0140-6736(16)31678-6 27733282 PMC5055577

[B64] Wang Y. Shen J. Chen Y. Liu H. Zhou H. Bai Z. (2018). PINK1 Protects against Oxidative Stress Induced Senescence of Human Nucleus Pulposus Cells via Regulating Mitophagy. Biochem. Biophysical Res. Commun. 504, 406–414. 10.1016/j.bbrc.2018.06.031 29890141

[B65] Xiong Y. Hannon G. J. Zhang H. Casso D. Kobayashi R. Beach D. (1993). p21 Is a Universal Inhibitor of Cyclin Kinases. Nature 366, 701–704. 10.1038/366701a0 8259214

[B66] Xue E. X. Lin J. P. Zhang Y. Sheng S. R. Liu H. X. Zhou Y. L. (2017). Pterostilbene Inhibits Inflammation and ROS Production in Chondrocytes by Activating Nrf2 Pathway. Oncotarget. Jun 27 (8), 41988–42000. 10.18632/oncotarget.16716 PMC5522043 28410217

[B67] Zheng W. Li X. Liu D. Li J. Yang S. Gao Z. (2019). Mechanical Loading Mitigates Osteoarthritis Symptoms by Regulating Endoplasmic Reticulum Stress and Autophagy. FASEB j. 33, 4077–4088. 10.1096/fj.201801851r 30485126 PMC6404578

