# Supplementary material for: Urolithin A Protects Chondrocytes From Mechanical Overloading-Induced Injuries
Source: Front Pharmacol. 2021 Jun 17;12:703847. doi: 10.3389/fphar.2021.703847 (PMC8245698; doi:10.3389/fphar.2021.703847)
Supplement: Supplementary file 1 [file DataSheet1.docx]

Supplementary Material

# Supplementary Tables

Table S1. Summary of donor information

| No. | Patient ID | Age | Sex | |
| --- | --- | --- | --- | --- |
| 1 | 2016152 | 73 | Female | Female Average  Age: 72.0 |
| 2 | 2018917 | 70 | Female |  |
| 3 | 20-0810-006 | 73 | Female |  |
| 4 | 2012529 | 69 | Male | Male Ave  Age: 69.3 |
| 5 | 2012473 | 70 | Male |  |
| 6 | 20-0818-006 | 69 | Male |  |

| Full name | Gene | Protein |
| --- | --- | --- |
| Ribosomal protein L13a | *RPL13a* | RPL13A |
| Collagen type II alpha 1 chain | *COL2* | COL2 |
| Aggrecan | *ACAN* | ACAN |
| SRY-Box transcription factor 9 | *SOX9* | SOX9 |
| Matrix metallopeptidase 13 | *MMP13* | MMP13 |
| Matrix metallopeptidase 1 | *MMP1* | MMP1 |
| Collagen type X | *COL10* | COL10 |
| Interleukin 6 | *IL6* | IL-6 |
| Interleukin 8 | *IL8* | IL-8 |
| Cyclin-dependent kinase inhibitor 2A | *CDKN2A* | p16 |
| Cyclin-dependent kinase inhibitor 1A | *CDKN1A* | p21 |
| Tumor antigen p53 | *TP53* | p53 |
| Lysosomal-associated membrane protein 1 | *LAMP1* | LAMP1 |
| Microtubule-associated proteins 1A/1B light chain 3B | *LC3B* | LC3B |
| PTEN-induced kinase 1 | *PINK1* | PINK1 |
| Parkin | *PARK2* | Parkin |
| Mitogen-activated protein kinase 1/2 | *p-ERK1/2* | p-ERK1/2 |
| Nuclear factor kappa-light-chain-enhancer of activated B cells | *NF-kB1* | NF-kB p65 |
| Caspase 3 | *CASP3* | CASP3 |
| BCL2-associated X protein | *BAX* | BAX |
| Nuclear factor erythroid 2-related factor 2 | *NRF2* | NRF2 |
| A disintegrin and metalloproteinase with thrombospondin motifs 5 | *ADAMTS5* | ADAMTS5 |
| Translocase of outer mitochondrial membrane 20 | *TOMM20* | TOM20 |
| Glyceraldehyde 3-phosphate dehydrogenase | *GAPDH* | GAPDH |

Table S2. Full name and abbreviation of gene and protein

Table S3. Primer sequences used for qRT-PCR.

| Gene | Forward primer (5’->3’) | Reverse primer (5’->3’) |
| --- | --- | --- |
| *RPL13a* | CATAGGAAGCTGGGAGCAAG | GCCCTCCAATCAGTCTTCTG |
| *COL2A1* | *GGATGGCTGCACGAAACATACCGG* | *CAAGAAGCAGACCGGCCCTATG* |
| *ACAN* | *AGTCACACCTGAGCAGCATC* | *AGTTCTCAAATTGCATGGGGTGTC* |
| *SOX9* | *GGCGGAGGAAGTCGGTGAAGAA* | *GCTCATGCCGGAGGAGGAGTGT* |
| *MMP13* | *ATGCAGTCTTTCTTCGGCTTAG* | *ATGCCATCGTGAAGTCTGGT* |
| *MMP1* | *AAAATTACACGCCAGATTTGCC* | *GGTGTGACATTACTCCAGAGTTG* |
| *COL10* | *CCCTCTTGTTAGTGCCAACC* | *AGATTCCAGTCCTTGGGTCA* |
| *ALP* | *ATCTTTGGTCTGGCCCCCATG* | *AGTCCACCATGGAGACATTCTCTC* |
| *OCN* | *TCACACTCCTCGCCCTATTG* | *GAAGAGGAAAGAAGGGTGCC* |
| *OPN* | *TCACCAGTCTGATGAGTCTCACCATTC* | *TAGCATCAGGGTACTGGATGTCAGGTC* |
| *IL-6* | *ACTCACCTCTTCAGAACGAATTG* | *CCATCTTTGGAAGGTTCAGGTTG* |
| *IL-8* | *ACTGAGAGTGATTGAGAGTGGAC* | *AACCCTCTGCACCCAGTTTTC* |
| *CDKN2A* | *CTACTGAGGAGCCAGCGTCT* | *CTGCCCATCATCATGACCT* |
| *CDKN1A* | *AGTGGAATTAGCCCTCAGCA* | *CATGGTCCCTGGGTTCTTC* |
| *TP53* | *GCCCAACAACACCAGCTCCT* | *CCTGGGCATCCTTGAGTTCC* |
| *ADAMTS5* | *GAACATCGACCAACTCTACTCCG* | *CAATGCCCACCGAACCATCT* |
| *NF-kB1* | *AACAGAGAGGATTTCGTTTCCG* | *TTTGACCTGAGGGTAAGACTTCT* |

Table S4. Information of primary antibodies used in this study

| Antibody | Company | Catalog number | Dilution |
| --- | --- | --- | --- |
| p21 | Abcam | ab218311 | IHC: 1:200; WB: 1:1000 |
| p16 | Abcam | ab108349 | IHC: 1:100; WB: 1:1000 |
| COL2A1 | Thermal Fisher | MA5-12789 | IHC: 1:100; WB: 1:100 |
| ACAN | Abcam | ab3778 | WB: 1:100 |
| SOX9 | Cell Signaling | D8G8H | WB: 1:1000 |
| LAMP1 | Thermal Fisher | PA1-654A | IHC: 1:50; WB: 1:500 |
| LC3B | Novus Biologicals | NB100-2220 | IHC: 1:200; WB: 1:1000 |
| PINK1 | MyBioSource | MBS3215961 | WB: 1:1000 |
| Parkin | Thermal Fisher | PA513399 | WB: 1:1000 |
| NRF2 | Novus Biologicals | NBP1-32822 | WB: 1:1000 |
| NF-κB p65 | Thermal Fisher | PA5-16545 | WB: 1:200 |
| BAX | Novus Biologicals | NB100-56095 | WB 1:1000 |
| p-Erk1/2 | Thermal Fisher | PA5-13036 | WB 1:1000 |
| CASP3 | Abcam | ab32042 | WB 1:500 |
| MMP13 | Abcam | ab39012 | WB 1:1000 |
| TOM20 | MyBioSource | MBS820677 | IF: 1:200; WB 1:1000 |
| p53 | Abcam | ab26 | WB 1:500 |
| GAPDH | Cell signaling | 5174 | WB: 1:1000 |
| Universal Kit | Vector | PK6200 | - |
| Mouse IgG | Invitrogen | 31450 | 1:1000 |
| Rabbit IgG | Healthcare | NA934-1ML | 1:1000 |

**Figure S1**


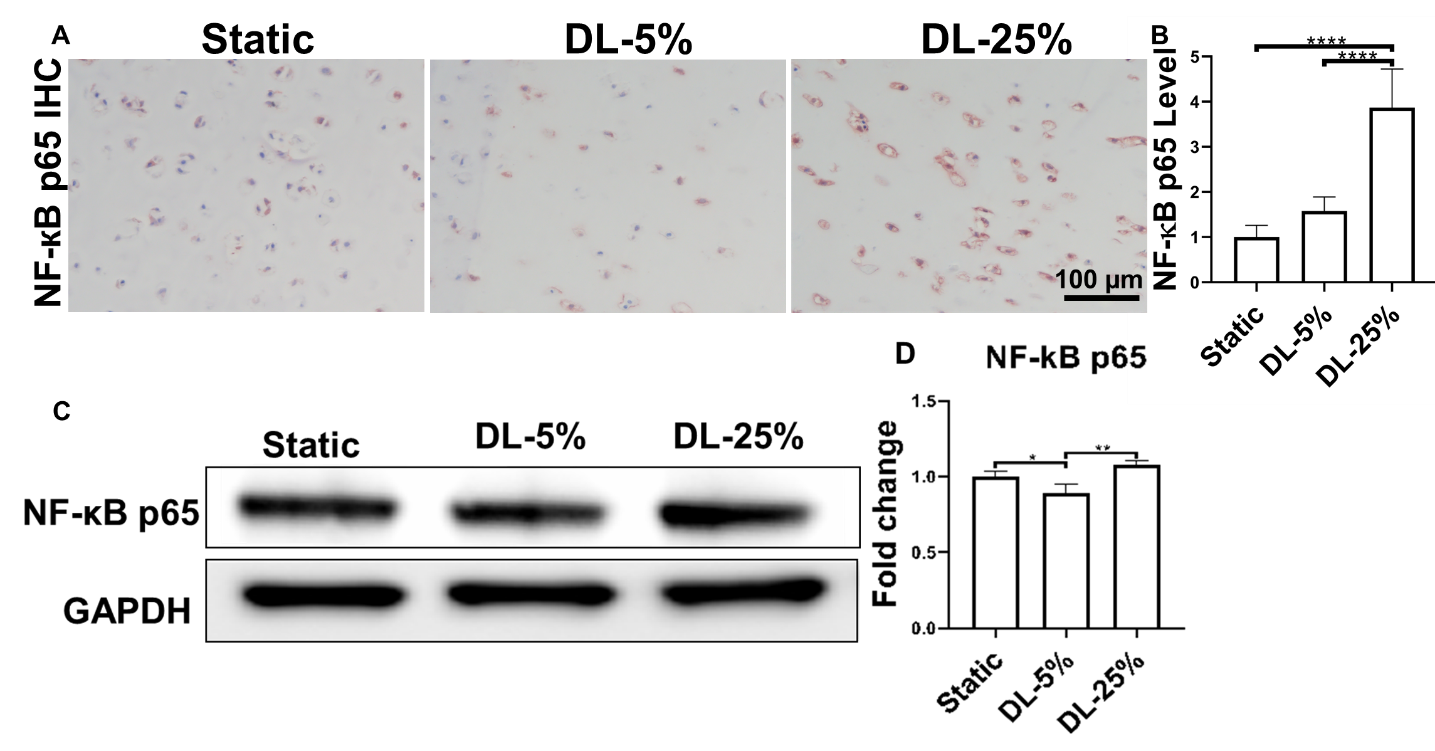


Figure S1. (A) NF-кB p65 IHC for samples from different groups. Scale bar: 100 µm. (B) Based on staining density, the level of NF-кB p65 were semi-quantitated using the ImageJ. (C) Representative western blot to assess the levels of NF-кB p65. (D) Semi-quantitative analysis of NF-кB p65protein level. N= 3. *, p<0.05; **, p<0.01; ****, p<0.0001.

**Figure S2**


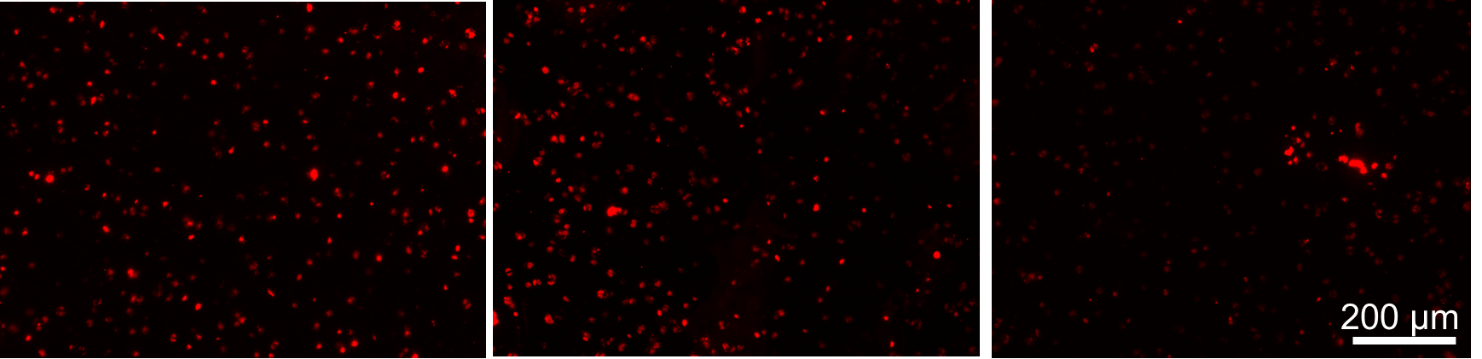


Figure S2. JC-1 immunofluorescence staining for samples from different groups. Scale bar: 100 µm.

**Figure S3**


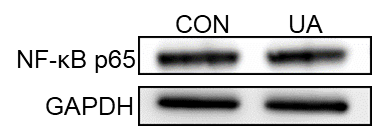


Figure S3. Representative western blot to assess the levels of NF-кB p65. CON: samples subjected to 25% strain loading; UA: samples subjected to 25% strain loading together with UA treatment (10 µM).
